# Genetic and epigenetic characterization of the BRCA1 gene in Brazilian women at-risk for hereditary breast cancer

**DOI:** 10.18632/oncotarget.13750

**Published:** 2016-12-01

**Authors:** Paula Silva Felicio, Matias Eliseo Melendez, Lidia Maria Rebolho Batista Arantes, Ligia Maria Kerr, Dirce Maria Carraro, Rebeca Silveira Grasel, Natalia Campacci, Cristovam Scapulatempo-Neto, Gabriela Carvalho Fernandes, Ana Carvalho de Carolina, Edenir Inêz Palmero

**Affiliations:** ^1^ Molecular Oncology Research Center, Barretos Cancer Hospital, Barretos, SP, Brazil; ^2^ Department of Pathology, Barretos Cancer Hospital, Barretos, SP, Brazil; ^3^ International Research Center, AC Camargo Cancer Center, São Paulo, SP, Brazil

**Keywords:** hereditary breast cancer, methylation, gene expression, breast cancer, BRCA1 gene

## Abstract

This study aimed to characterize women at-risk for hereditary BC regarding their clinical and molecular characteristics (mutation and methylation in the *BRCA1* gene) and correlate the gene expression levels with histopathological, clinical and family history information. *BRCA1* real time qPCR was performed to evaluate methylation status and gene expression. The study included 88 women grouped according to the *BRCA1* mutational status: 23 *BRCA1* mutated, 22 with a Variant of Unknown Significance (VUS) in *BRCA1* and 43 *BRCA1* WT. Most *BRCA1* mutated tumors were triple negative (69.6%) and had histologic grade III (61.0%). Patients with VUS/WT *BRCA1* were predominantly of luminal B subtype with histological grades I and II. Regarding the methylation profile, *BRCA1* hypermethylation was observed in only two patients (both WT) and none had association with pathogenic *BRCA1* mutation. In one patient methylation was present in both, tumor and normal tissues. Hypermethylated tumors had ductal histology, negativity for ER and occurred in < 50 years patients. Gene expression profile showed in all groups lower *BRCA1* mRNA levels in tumor tissue compared to the adjacent breast tissue, thereby indicating the loss/decrease of gene function. No association was found between the levels of *BRCA1* gene expression and family history of cancer. In summary, our findings suggested that methylation at the *BRCA1* gene is not the “second” event in the development of BC in patients with germline mutations in *BRCA1* and, although rare, *BRCA1* epimutations can constitute an explanation for a fraction of HBOC families.

## INTRODUCTION

Breast cancer (BC) is the most common malignancy in women, accounting for 22% of new cases of cancer each year [1]. It is estimated that for BC, as known for most malignant tumors, 5 to 10% are hereditary [2]. Germline mutations in the tumor suppressor genes, *BRCA1* and *BRCA2* account for approximately 20% of cases of hereditary breast cancer cases [3]. Furthermore, germline mutation in *BRCA1* gene carriers have a cumulative risk of developing breast cancer ranging from 44% to 68% by 70 years of age [4]. Mutations in *BRCA1* and *BRCA2* genes are associated with the Hereditary Breast and Ovarian Cancer Predisposition Syndrome (HBOC). Patients who have HBOC syndrome have a personal and a strong family history of cancer mainly in the following organs: breast, ovarian, pancreas and prostate. HBOC families, like other families with hereditary cancer predisposition syndromes, are characterized by early age on diagnosis, multiple primary tumors, bilateral tumors or multiple rare tumors and two or more generations affected by cancer [5, 6].

*BRCA1* and *BRCA2* are involved in the maintenance of genomic integrity through various cellular processes such as DNA damage recognition, transcription and cell cycle regulations and repair of DNA damage [7]. Given this, pathological alterations in these genes may cause changes in the function of its proteins. To date more than 2,500 proved pathogenic mutations have been described throughout the coding sequence *BRCA1* and *BRCA2*, which can be found in the database ClinVar [8] as well as in the Biobase portal - HGMD (Human Genome Mutation Database) [9]. In addition to genetic alterations, the occurrence of epigenetics alterations, such as hypermethylation of tumor suppressor genes, could be responsible for silencing this gene and consequently increase the risk of cancer development [10, 11].

The evaluation of the status of gene promoter methylation has been considered a potential biomarker for various tumor types and an useful tool for tumor detection or as a prognostic factor, as described for many different cancers such as lung cancers [12], colorectal [13], head and neck [14], and even for breast tumors [15–18].

Although the correlation between promoter hypermethylation of *BRCA1* gene with transcriptional inactivation has been reported in several studies [19, 20], as well as the association of the methylation level with clinical stage [20], histologic grade [20], triple negative phenotype [20, 21], and ancestry [21], there is little evidence on the correlation between the *BRCA1* methylation status and hereditary breast cancer. The methylation profile of mutation carriers was first described in 2001 by Esteller *et al*., who proposed that in hereditary tumors caused by alterations in tumor suppressor genes, the importance of inactivation by epigenetic events depends on which genes are involved in the tumor under study. According to the author, carriers of germline *BRCA1* mutations present a higher frequency of genetic events (and not epigenetic) as the “second event”.

In this study, our objective was to characterize women at-risk for hereditary BC regarding their status of *BRCA1* mutation and methylation and to correlate these results with the levels of gene expression, histopathological and clinical data, as well as with prognosis and family history of cancer.

## RESULTS

### Patient and tumor characteristics

The average age at diagnosis of the patients included was 39.0 years (SD = 9.4). When patients are grouped according to germline *BRCA1* mutational status, the mean age at diagnosis for women of the *BRCA1*-pathogenic groups, *BRCA1*-VUS and *BRCA1*-WT were respectively 41.6; 37.2 and 36.5 years (SD = 7.3, 9.4 and 10.1 years, respectively).

Additionally, the age at diagnosis was categorized and the information, as well as the clinical and pathological data of the cases included in the study, are detailed in Table 1.

**Table 1:** Clinicopathological characterization of the patients

		*BRCA1*-pathogenic	*BRCA1*-VUS	*BRCA1*-WT	*p*-value
		*n* = 23	*n* = 22	*n* = 43	
**Age at diagnosis**	≤ 30 years	0 (0.0%)	7 (31.8%)	10 (23.3%)	
	> 30 and ≤ 50 years	21 (91.3%)	12 (54.5%)	27 (62.8%)	0.020**
	> 50 years	2 (8.7%)	3 (13.6%)	6 (14.0%)	
**Bilateral tumor**	No	13 (56.5%)	15 (68.2%)	33 (76.7%)	0.247*
	Yes	10 (43.5%)	7 (31.8%)	10 (23.3%)	
**Histological type**	Ductal/lobular carcinoma “*in situ*”	4 (17.4%)	2 (9.1%)	4 (9.3%)	0.676**
	Invasive ductal/lobular carcinoma	19 (82.6%)	20 (90.9%)	39 (90.7%)	
**Histological grade**	I	1 (4.3%)	1 (4.5%)	7 (16.2%)	
	II	4 (17.4%)	11 (50.0%)	15 (34.9%)	0.072**
	III	14 (61.0%)	7 (31.9%)	17 (39.6%)	
	Unknown	4 (17.3%)	3 (13.6%)	4 (9.3%)	
**Clinical staging**	0 or I	5 (26.3%)	6 (37.5%)	18 (48.6%)	
	II	9 (47.4%)	6 (37.5%)	15 (40.5%)	
	III	5 (26.3%)	4 (25.0%)	3 (8.1%)	0.332**
	IV	0 (0.0%)	0 (0.0%)	1 (2.7%)	
**Tumor size**	T1	7 (30.4%)	8 (36.4%)	20 (46.5%)	0.415*
	T2-T4	16 (69.6%)	14 (63.6%)	23 (53.5%)	
**Lymph node status**	N0	11 (47.8%)	13 (59.1%)	28 (65.1%)	0.396*
	N1-N3	12 (52.2%)	9 (40.9%)	15 (34.9%)	
**Distant metastasis**	M0	23 (100%)	22 (100%)	40 (93.0%)	0.431**
	M1	0 (0.0%)	0 (0.0%)	3 (7.0%)	

* Chi-square test

** Fisher's Exact Test.

Regarding hormone receptors, estrogen (ER) and progesterone (PR) were predominantly negative in the *BRCA1*-pathogenic group, unlike that observed in the *BRCA1*-VUS and *BRCA1*-WT groups (*p* = 0.008 and *p* = 0.003, respectively; Table 2). On the other side, the negativity of the human epidermal growth factor receptor-type 2 (HER2) was frequent in all three groups. Thirty patients were triple negative, with 53.3% of them carrying *BRCA1* pathogenic mutations, 16.7% with a VUS on *BRCA1* and 30% were *BRCA1* WT (*p* < 0.005).

**Table 2:** Expression of hormone receptors, HER2 and Ki67

		*BRCA1*-pathogenic	*BRCA1*-VUS	*BRCA1*-WT	*p*-value
		*n* = 23	*n* = 22	*n* = 43	
**Estrogen receptor (ER)**	Negative	16 (69.6%)	7 (31.8%)	14 (32.6%)	0.008*
	Positive	7 (30.4%)	15 (68.2%)	29 (67,4%)	
**Progesterone receptor (PR)**	Negative	19 (82.6%)	8 (36.4%)	19 (44,2%)	0.003*
	Positive	4 (17.4%)	14 (63.6%)	24 (55,8%)	
**HER-2**	Negative	20 (90.9%)	17 (85.0%)	32 (74,4%)	0.270**
	Positive	2 (9.1%)	3 (15.0%)	11 (25,6%)	
**Ki67**	≤ 14%	2 (8.7%)	2 (9.0%)	4 (9.3%)	
	> 14%	14 (60.9%)	16 (72.8%)	32 (74.5%)	1.000*
	Unknown	7 (30.4%)	4 (18.2%)	7 (16.2%)	

* Chi-square test

** Fisher's Exact Test.

### Family history

To obtain the family history of cancer, the pedigree of the 88 families included in the study were reviewed. In this analysis, information regarding the presence, frequency and age at diagnosis of primary tumors related to HBOC were considered, such as the presence of breast cancer (female and male), ovary, pancreas and prostate. The average age at breast cancer diagnosis in the family was 42.6 (SD = 9.4). When the three groups were considered separately, the average age at diagnosis of breast cancer in the family, in the *BRCA1*-pathogenic, *BRCA1*-VUS and *BRCA1*-WT groups were respectively 44.1; 41.2 and 42.5 years (SD = 5.7, 9.7 and 10.7 years, respectively).

Table 3 depicts the family history in relation to the *BRCA1* germline mutational status. We observed a higher proportion of reports of breast cancer before the age of 50 in families with pathogenic *BRCA1* mutations (*p* = 0.005). In addition, presence of breast cancer among mother and daughter was seen in all three groups, with a greater propensity for such phenomenon among patients in the *BRCA1*-pathogenic group (*p* = 0.055). The total number of breast cancer cases in the family was also evaluated. As expected, the majority of *BRCA1* mutated patients had 3 or more cases of breast cancer in their families (73.9%), while most patients from *BRCA1*-VUS and *BRCA1*-WT groups (63.6% and 64.3%) reported up to two cases of breast cancer in the family (*p* = 0.007).

**Table 3:** Family history according to the BRCA1 mutational status

		*BRCA1*-pathogenic	*BRCA1*-VUS	*BRCA1*-WT
		*n* = 23	*n* = 22	*n* =4 3
**Presence of bilateral breast cancer**	No	13 (56.5%)	15 (68.2%)	33 (76.7%)
	Yes	10 (43.5%)	7 (31.8%)	10 (23.3%)
	*p*-value	0.235*		
**Presence of pancreatic cancer**	No	21 (91,3%)	20 (90.9%)	42 (97.7%)
	Yes	2 (8,7%)	2 (9.1%)	1 (2.3%)
	*p*-value	0.296**		
**Presence of ovarian cancer**	No	16 (69.6%)	18 (81.8%)	41 (95.3%)
	Yes	7 (30.4%)	4 (18.2%)	2 (4.7%)
	*p*-value	0.015**		
**Presence of breast and ovarian cancer**	No	20 (87.0%)	21 (95.5%)	43 (100.0%)
	Yes	3 (13.0%)	1 (4.5%)	0 (0.0%)
	*p*-value	0.039**		
**Presence of male breast cancer**	No	23 (100.0%)	22 (100.0%)	42 (97.7%)
	Yes	0 (0.0%)	0 (0.0%)	1 (2.3%)
	*p*-value	1.000**		
**Presence of prostate cancer**	No	20 (87.0%)	15 (68.2%)	35 (81.4%)
	Yes	3 (13.0%)	7 (31.8%)	8 (18.6%)
	*p*-value	0.300**		
**Presence of breast cancer among mother and daughter**	No	12 (52.2%)	17 (77.3%)	34 (79.1%)
	Yes	11 (47.8%)	5 (22.7%)	9 (20.9%)
	*p*-value	0.055*		
**Breast cancer cases < 50 years**	0	0 (0.0%)	2 (9.1%)	3 (7.0%)
	1–2	12 (52.2%)	18 (81.8%)	35 (81.4%)
	≥ 3	11 (47.8%)	2 (9.1%)	5 (11.6%)
	*p*-value	0.005**		
**Generations affected by breast cancer**	1–2	21 (91.3%)	20 (90.9%)	38 (88.4%)
	≥ 3	2 (8.7%)	2 (9.1%)	5 (11.6%)
	*p*-value	1.000**		
**Total number of breast cancer cases in the family**	1–2	6 (26.1%)	14 (63.6%)	27 (64.3%)
	≥ 3	17 (73.9%)	8 (36.4%)	15 (35.7%)
	*p*-value	0.007**		

* Chi-square test

** Fisher's Exact Test.

### Methylation analysis

**Figure 1:**
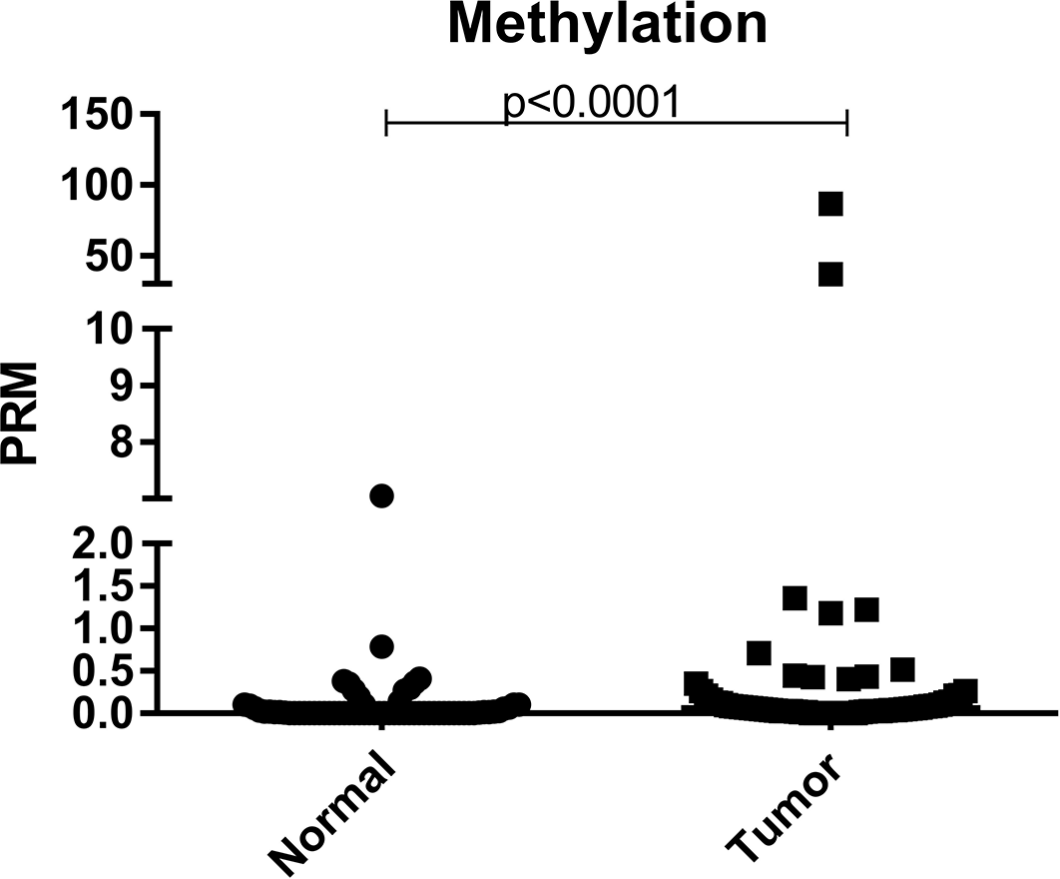
Scatter plot of the percentage of relative methylation (PRM) of the samples analyzed in the study Left: normal samples and right: tumor samples. The Y-axis shows the PMR level. The *p*-value (Mann-Whitney) from each comparison is provided.

First, the Percentage of Relative Methylation (PRM) between all normal and tumor samples was compared independently using the Mann-Whitney test, in order to check if there is difference in the level of methylation profile between these two groups. Through this analysis, it was observed that there is a statistically significant difference in the profile of methylation in the promoter region of *BRCA1* between normal and tumor tissues, with a higher level of methylation being observed in tumor samples when comparing to normal samples (*p* = 0.0001) (Figure 1).

**Figure 2:**
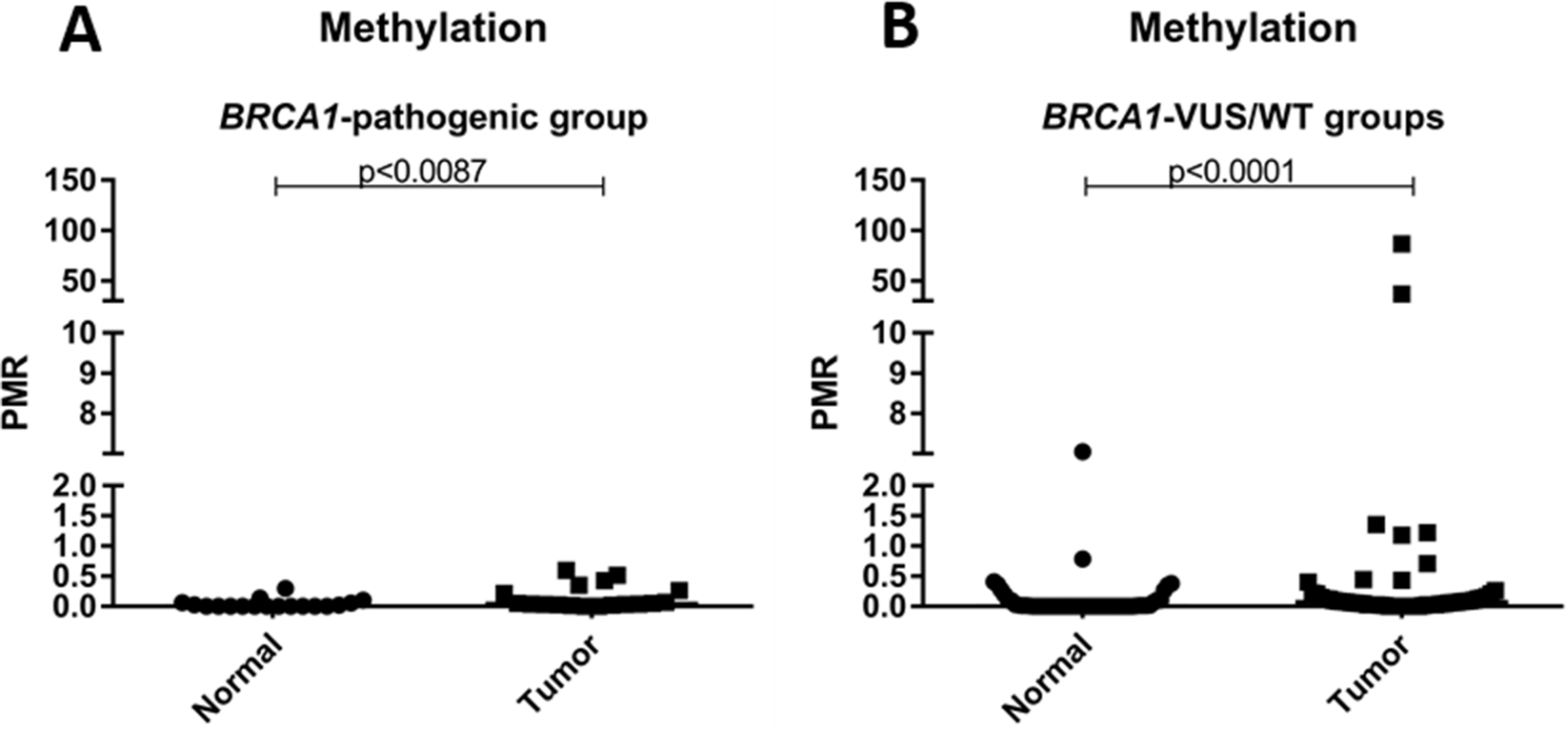
Scatter plot of the percentage of relative methylation (PRM) of normal and tumor samples according to mutation status (**A**) PRM representation only in patients with deleterious germline mutation in *BRCA1* (*BRCA1*-pathogenic group). (**B**) PRM representation only in patients without deleterious germline mutation in *BRCA1* (*BRCA1*-VUS groups and *BRCA1*-WT). The Y-axis shows the PMR level. The *p*-value (Mann-Whitney) from each comparison is provided.

Secondly, we compare the level of methylation of mutated versus non-mutated patients, and no difference was observed between the groups (*p* = 1.000). Additionally, the level of methylation of normal and tumor samples was analyzed according to the mutational status of *BRCA1* (mutated vs. non-mutated). No statistical difference was observed when comparing tumor and normal samples in the mutated group (*p* = 0.0097), however, this comparison was significant in the non-mutated group. The results of this analysis are shown in Figure 2.

In the sequence, to classify the samples as methylated or unmethylated, a cut-off of 4% was set, and, using this stratification, *BRCA1* promoter hypermethylation was found in 2 of 88 (2.3%) breast tumor samples analyzed. When the methylation status was evaluated according to the *BRCA1* mutational status, patients with deleterious germline mutation showed no hypermethylation of the *BRCA1* gene, whereas 2/43 patients of the *BRCA1*-WT group showed methylation in the promoter region of the *BRCA1* gene. An interesting finding was the identification of *BRCA1* hypermethylation in the normal adjacent tissue in one sample (PRM = 37% in the tumor tissue and 7% in the normal tissue). To eliminate the possibility of a contamination from tumor cells, a blood sample from that patient was investigated and the *BRCA1* hypermethylation was verified (PRM = 9%), confirming the presence of a constitutive *BRCA1* epimutation. The PRM of the second methylated sample was 86.7%, however for this sample only the tumor tissue was hypermethylated.

### Gene expression analyses

The evaluation of *BRCA1* expression was performed by RT-qPCR for 68 tumor samples and 49 matched normal samples (54 samples were excluded due to poor RNA quality). Among the tumor samples, 16/68 (23.5%) belonged to the *BRCA1*-pathogenic group (mutated *BRCA1*), 16/68 (23.5%) were from *BRCA1*-VUS group and 36/68 (53%) were *BRCA1* WT. Regarding normal samples, 11/49 (22.5%) belonged to the *BRCA1*-pathogenic group, 12/49 (24.5%) to the *BRCA1*-VUS group and 26/49 (53.0%) were *BRCA1* WT.

**Figure 3:**
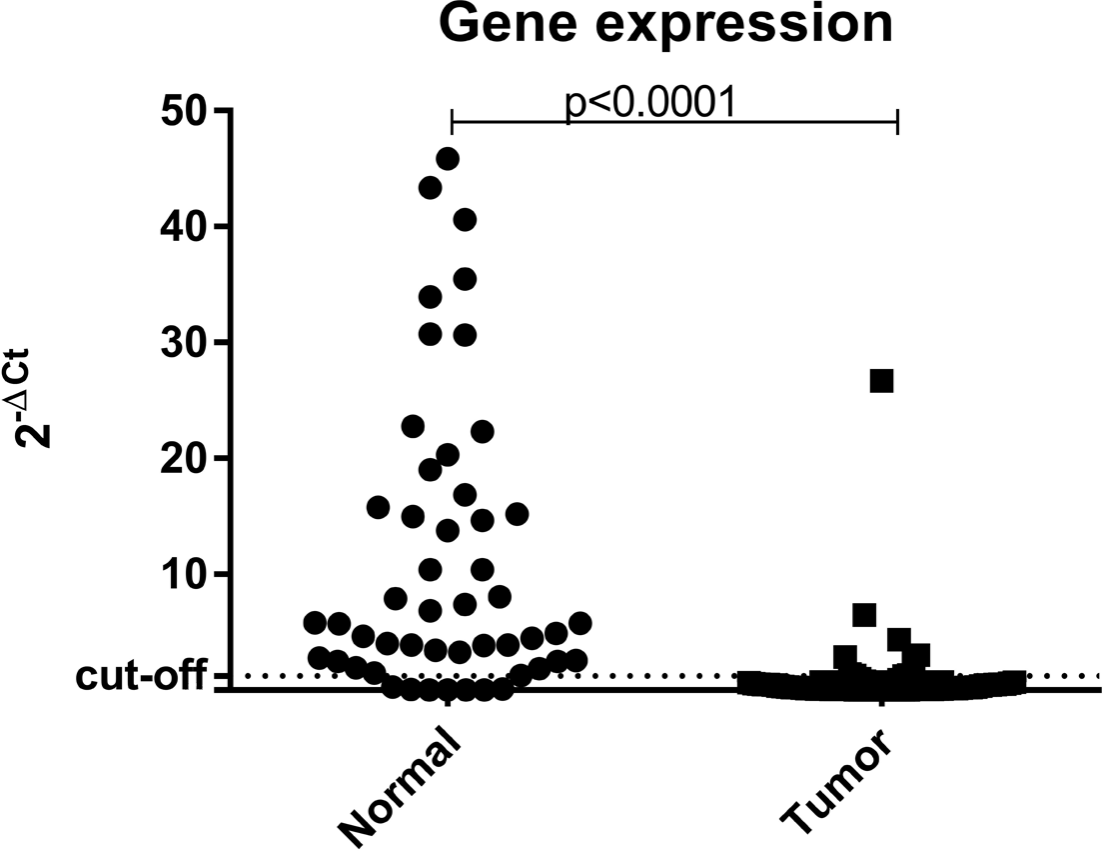
Scatter plot of the expression profile of BRCA1 in normal and tumor samples suitable for analysis The Y-axis shows the fold-change of the relative expression (2^-ΔCt^). The *p*-value (Mann-Whitney) from each comparison is provided. The dotted line indicates the median value used as cutoff. Left: normal samples. Right: tumor samples.

The Mann-Whitney test showed a statistically significant difference (*p* < 0.0001) between the gene expression of normal samples vs tumor samples (Figure 3). In addition, when using the ΔCT method to estimate the level of expression and the median value of all samples evaluated as the cutoff point, all normal samples analyzed showed high levels of *BRCA1* expression. Unfortunately, gene expression analysis could not be performed on the sample with constitutive *BRCA1* epimutation due to low RNA quality. However, if only one endogenous gene is used as a reference for the assessment of gene expression, we note that those tumor samples showed low *BRCA1* mRNA expression.

**Figure 4:**
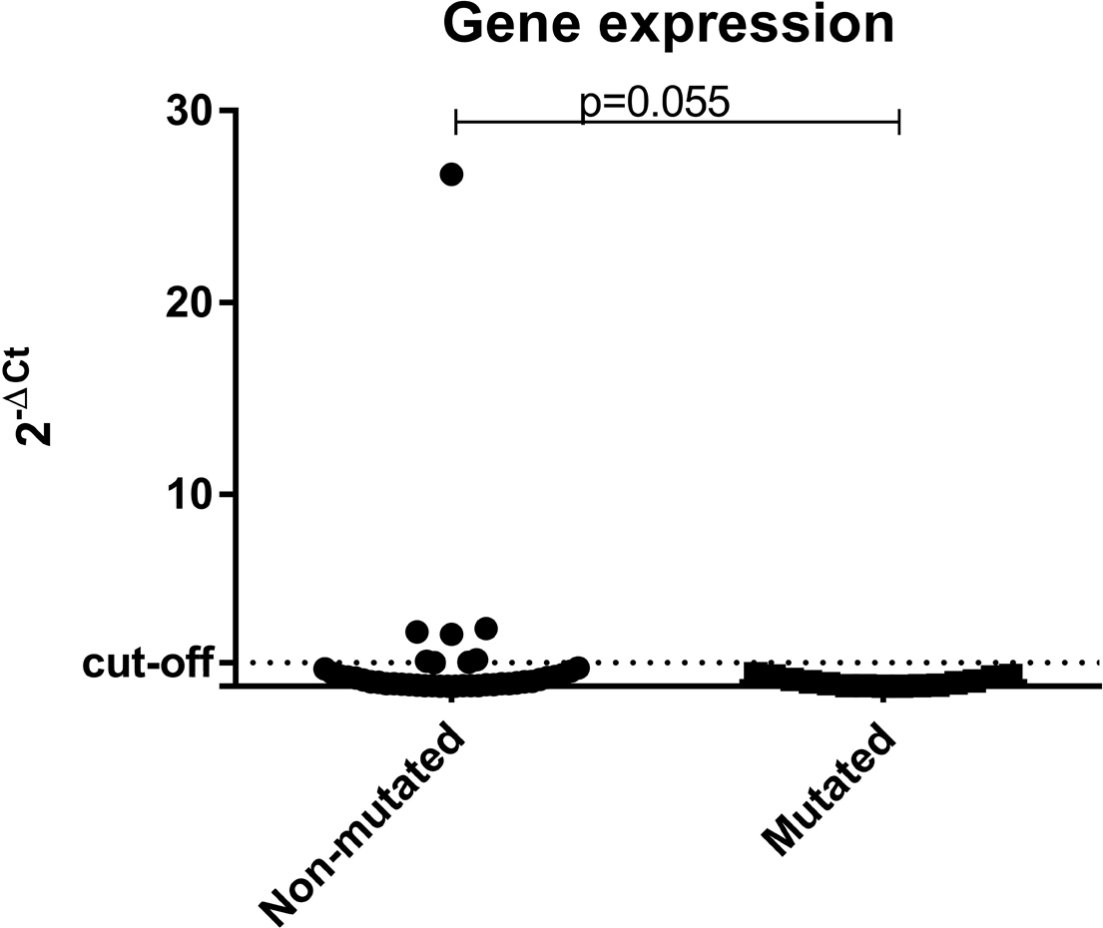
Scatter plot of the expression profile of BRCA1 in tumor samples according to the status of BRCA1 germline mutation The Y-axis shows the fold-change of the relative expression (2^-ΔCt^). The *p*-value (Mann-Whitney) from each comparison is provided. The dotted line indicates the median value used as cutoff. Left: patients without deleterious *BRCA1* germline mutation (*BRCA1*-VUS and *BRCA1*-WT groups). Right: patients with deleterious germline mutation in *BRCA1* (*BRCA1*-pathogenic group).

The great majority of the tumor samples analyzed, (57/68; 83.8%) had low expression levels of *BRCA1*. When comparing gene expression results among the three groups separately, low levels of *BRCA1*could be observed in all patients (16/16; 100%) with deleterious germline mutation in *BRCA1*, in 11/16 (68.8%) of *BRCA1*-VUS patients, and 30/36 (83.3%) of those WT for *BRCA1*. When patients were categorized into only two groups (non-mutated vs. mutated) it was observed that 16/16 (100%) patients with deleterious germline mutation in *BRCA1* have low gene expression levels in tumor samples, as well as 41/52 (78.8%) of those patients without deleterious germline mutation (*BRCA1*-VUS and *BRCA1*-WT groups) (Figure 4).

### Association between clinicopathologic, molecular and family history features

The expression levels of *BRCA1* in patients with *BRCA1* hypermethylation could not be evaluated due to poor RNA quality.

**Figure 5:**
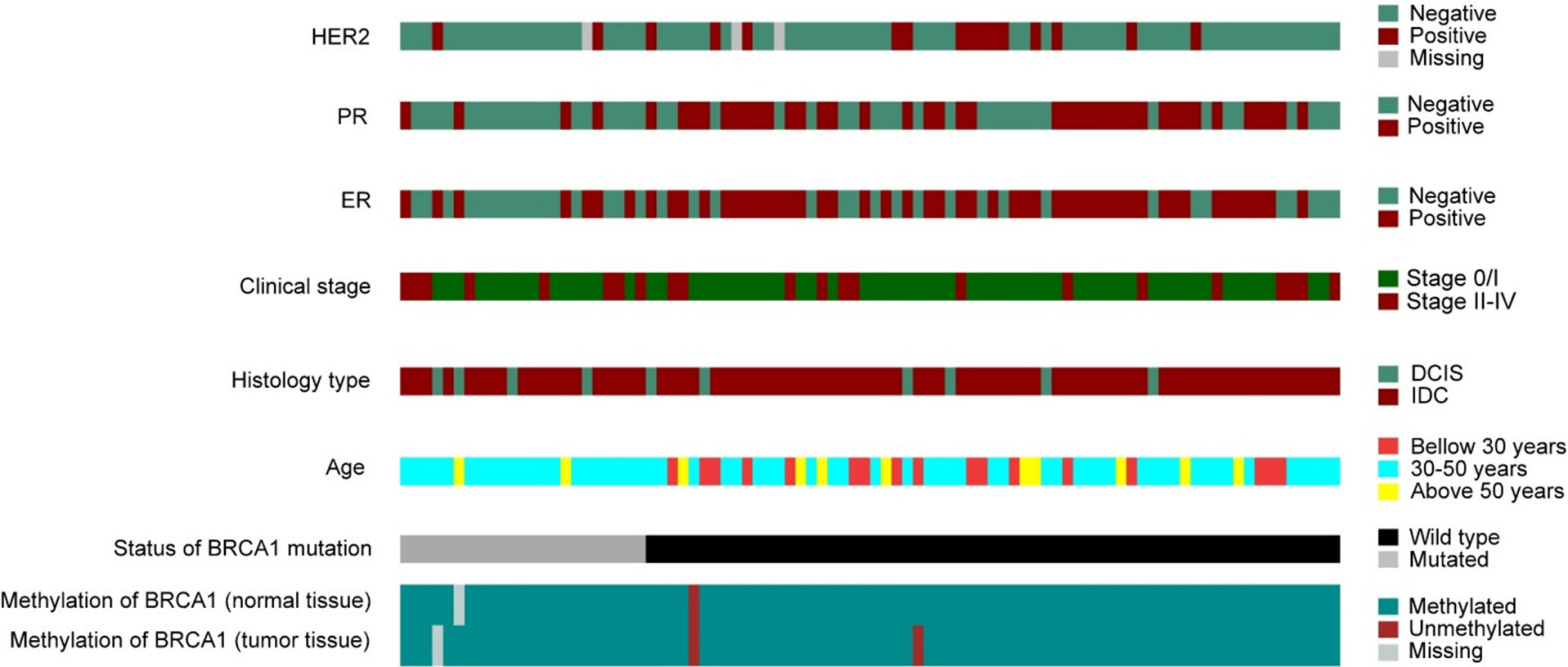
Molecular and pathological characteristics (rows) of the patients included in the study Patients are organized in columns and arranged to emphasize mutual exclusivity among characteristics.

The description of the clinical and molecular data is shown in Figure 5. The presence of an association between the level of methylation and clinical and histopathologic variables was analyzed and the results of this analysis are described in Table 4. We did not find any statistically significant association among the variables, possibly due to the small number of methylated samples. Both cases with *BRCA1* hypermethylation had invasive ductal tumors and were negative for the estrogen receptor. Moreover, the breast cancer developed by the patient carrying the epimutation was bilateral and diagnosed at 37 years of age.

**Table 4:** Clinical and histopathological characteristics compared to the methylation profile

		Methylated	Non-Methylated	
		*n* = 2	*n* = 85	*p*-value
**Germline deleterious mutation**	No	2 (100%)	63 (74.1%)	1.000**
	Yes	0 (0.0%)	22 (25.9%)	
**Histological type**	Invasive ductal/lobular carcinoma	2 (100%)	76 (89.4%)	1.000**
	Ductal/lobular carcinoma *in situ*	0 (0.0%)	9 (10.6%)	
**Estrogen receptor**	Negative	2 (100%)	35 (41.2%)	0.178**
	Positive	0 (0.0%)	50 (58.8%)	
**Progesterone receptor**	Negative	1 (50.0%)	44 (51.8%)	1.000**
	Positive	1 (50.0%)	41 (48.2%)	
**HER2**	Negative	2 (100%)	67 (78.9%)	
	Positive	0 (0.0%)	15 (17.6%)	1.000**
	Unknown	0 (0.0%)	3 (3.5%)	
**Triple Negative**	No	1 (50.0%)	56 (65.9%)	1.000**
	Yes	1 (50.0%)	29 (34.1%)	

** Fisher's Exact Test.

Although no significant correlation between the methylation profile and clinical and histopathological features was found, we can highlight that none patient with deleterious germline mutation in *BRCA1* showed hypermethylation. In addition, patients with methylation in the *BRCA1* gene, showed negativity for the estrogen receptor (Figure 5).

By correlating the expression levels of *BRCA1* with clinical and tumor characteristics we observed that a significant proportion of cases with high levels of *BRCA1* expression (35.0%) had tumors at early stages (T1, N0 and M0). Among those less advanced and with low gene expression tumors (T1), the majority (76.2%, 16/21) had luminal subtype, followed by 19.0% (4/21) triple negatives and 4.8% (1/21) were HER2 subtype. In addition to the histopathological data, we evaluated the association between *BRCA1* transcriptional levels with the status of hormone receptors (ER and PR), HER2 and Ki67. Although the relationship between transcriptional level and hormone receptors status was not significant, it can be observed that most of the triple negative patients (20/22) presented low *BRCA1* expression, whereas patients with high expression were mostly Luminal (Table 5).

**Table 5:** Association between gene expression and molecular subtype

		Low Expression	High Expression	*p*-value
		*n* = 57	*n* = 11	
**Molecular Subtype**	Luminal (Luminal A, Luminal B)	33 (57.9%)	7 (63.6%)	
	HER2 (ER−, PR−, HER2+)	1 (1.7%)	2 (18.2%)	0.049**
	Triple-Negative (ER−, PR−, HER2−)	20 (35.2%)	2 (18.2%)	
	Unknown	3 (5.2%)	0 (0.0%)	

* Fisher's Exact Test.

Values in bold indicate statistical significance (*p* < 0.05).

Regarding the family history, the average age at diagnosis of breast cancer in the family in the three groups was 42.6 years (SD = 9.4). When the three groups are considered separately, the average age at diagnosis of breast cancer in the family, in the *BRCA1*-pathogenic, *BRCA1*-VUS and *BRCA1*-WT groups were respectively 44.1; 41.2 and 42.5 years (SD = 5.7, 9.7 and 10.7 years, respectively).

We observed a higher proportion of breast cancer cases younger than 50 years in families with pathogenic mutations in *BRCA1*, while most patients the *BRCA1*-VUS and *BRCA1*-WT groups reported only one or two cases diagnosed before the age of 50 (*p* = 0.005). Besides, 73.9% of the families in the *BRCA1*-pathogenic group had 3 or more cases of breast cancer in their families, while 63.6% and 64.3% of the families with VUS at *BRCA1* gene and *BRCA1*-WT respectively, reported up to two cases of breast cancer in the family (*p* = 0.007). Furthermore, when we look at the family history of the patient who presented the epimutation, we can highlight the presence of two prostate tumors and only one generation affected by breast cancer.

## DISCUSSION

The identification of individuals and families with hereditary cancer is important because affected individuals have a vital cumulative risk much higher than the population for the development of various cancers. These tumors usually occur at younger ages than it would be expected for the pathology in question. This study was conducted in order to characterize women with personal and family history of breast cancer (with and without germline mutation in *BRCA1*) regarding their clinical and molecular characteristics (mutation and methylation in *BRCA1*). The transcriptional level of *BRCA1* was also tested for possible associations with pathological and morphological characteristics of breast cancer, as well as clinical characteristics, prognosis and family history.

The inclusion was based on the mutational status of *BRCA1* (mutated, with a VUS or WT) and no patient harboring a *BRCA2* mutation was included. The majority of patients (69.6%) with a pathogenic mutation at *BRCA1* were triple negative (as expected). Conversely patients with a VUS on *BRCA1* and those harboring WT *BRCA1* had a similar proportion of triple negative tumors (31.8% and 32.6% respectively), a percentage slightly higher than that observed in sporadic cases of breast cancer [22].

Several studies have reported the association of triple negative breast cancer with the presence of pathogenic mutations in *BRCA1*. A study published by the CIMBA group reported a frequency of 69% of TN tumors in 3,797 patients with *BRCA1* mutations [23]. Similarly, a study published by Carraro *et al*. showed a frequency of 71.4% of TN tumors among a small cohort of patients with germline *BRCA1* mutation and early diagnosis of breast cancer [24].

The higher prevalence of TN cases among *BRCA1* mutated patients can be one of the factors responsible for the poor prognosis observed in these patients. Studies point to the fact that tumors associated with the presence of *BRCA1* mutations often have higher histologic grade, and elevated mitotic counts, are poorly differentiated, with high frequency of necrotic areas and pleomorphism. These characteristics are commonly associated with a worse prognosis [23, 25, 26]. In our study, we observed that most patients with mutation in *BRCA1* showed histological grade III (61.0%), triple negativity and higher cell proliferation index.

Besides having a tumor profile characteristic of tumors with a poor prognosis, the family history associated with those carrying a pathogenic *BRCA1* mutation was more severe than the WT or with a VUS, as it would be expected. The mean age at cancer diagnosis in the *BRCA1*-pathogenic group was 41.6 years, in the VUS-*BRCA1* group this value decreased to 37.2 years, and for the WT-*BRCA1* group the mean age was 36.5 years. This finding points to the fact that maybe most of those families were referred by genetic testing due to an earlier age at cancer diagnosis and not due to a strong family history of cancer. Although the presence of mutations in other breast and ovarian predisposition genes can be the explanation for the cancer cases identified in the WT and VUS groups, this was not investigated in the present study. The subsequent search for a better understanding of the characteristics of families at-risk for breast cancer, as well as to more accurately determine the risk of cancer is critical. Knowing which gene is altered and the mechanisms associated with this change makes possible to expand the range of preventive strategies to be offered.

Other than germline mutation status, we also investigated the methylation profile of *BRCA1* in breast tumor and normal counterpart. The presence of methylation in the promoter region of *BRCA1* in sporadic breast tumors has been reported in several studies. A study conducted in 2000 by Rice et al., detected the presence of hypermethylation in 3/21 breast tumors with concomitant lower levels of mRNA. The authors suggested that epigenetic silencing of *BRCA1* can be a transcriptional inactivation mechanism responsible for the development of mammary tumorigenesis in sporadic cases [27]. A more recent study, conducted by Hsu et al., analyzed the methylation profile of 139 patients diagnosed with breast cancer at an early stage. The authors reported the presence of hypermethylation in 56% of the analyzed tumors and this alteration was correlated with the occurrence of triple negative tumors [21]. In 2014, work by Sharma and colleagues reported the presence of *BRCA1* methylation in 30% of women with sporadic breast cancer [28]. However, studies involving methylation analysis in tumor tissue of patients carriers of a *BRCA1* germline pathogenic mutation are scarce [29–31].

Although well known for hereditary colorectal tumors [17, 32], the presence of epigenetic mutations in hereditary breast cancer is not well understood. Results published by Dworkin et al., in 2009 reaffirmed the proposed by Esteller in 2001, that the hypermethylation of the *BRCA1* promoter region is not the most frequent “second-hit” in patients with germline mutation in this gene [29]. Recently, Lips et al., found that none of the tumors analyzed in their study presented both events: germline mutation and methylation in the *BRCA1* gene. These data suggest that *BRCA1* is only inactivated by genetic or epigenetic events [30].

In the present study we identified two patients with hypermethylation of the *BRCA1* gene, one of those with a constitutive epimutation (confirmed in DNA obtained from peripheric blood). That patient was *BRCA1* and *BRCA2* WT and had a personal history of bilateral breast cancer (diagnosed at 37 and 41 years old), both tumors were invasive ductal carcinomas with estrogen receptor negative. Moreover, in line with the cited studies, none of the analyzed patients had both events: germline mutation in *BRCA1* and hypermethylation. In 2014, Al-Moghrabi *et al*. investigated the presence of constitutive *BRCA1* hypermethylation in 155 breast cancer patients and 143 cancer-free females and found that constitutive epimutation can constitute a mechanism for breast cancer predisposition, being present in 14.2% of breast cancer patients and 9.1% of cancer-free females (with a positive family history of cancer) [33].

The other patient with *BRCA1* hipermethylated identified in our study also presented negativity for the estrogen receptor and was *BRCA1* WT. Study conducted by Birgisdottir *et al*. showed that the hypermethylation of *BRCA1* in sporadic tumors was associated with the loss of estrogen receptor expression. Moreover, it was present in a higher frequency in women diagnosed under the age of 50, suggesting that the tumors with aberrant methylation in *BRCA1* are similar to tumors with pathogenic mutation in *BRCA1*, leading to the same *BRCA*-ness phenomenon [34].

In our study, due to the poor quality of a proportion of RNA samples extracted from FFPE for the analysis of gene expression, it was not possible to verify the expression levels of *BRCA1* of patients with hypermethylation. Furthermore, we demonstrate that the profile of *BRCA1* gene expression was significantly different between normal vs. tumor tissue.

An interesting fact observed was that a significant number of cases, which did not have germline mutations nor methylation in *BRCA1* had low levels of *BRCA1* gene expression, leading us to speculate which could be the mechanisms related to this silencing. Among the mechanisms that may be involved, one could emphasize the presence of somatic *BRCA1* mutation, LOH, mRNA decay, miRNA regulation, among others. Moreover, we cannot exclude the possibility of methylation in the “body of the gene” or in other regions of the promoter not covered in this study. Thus, considering the findings of this study and the possible mechanisms involved, further studies involving LOH analysis, miRNA, methylation in other regions of the promoter or the gene body, as well as protein analysis and functional studies should be conducted to analyze in detail the reason why the expression levels of *BRCA1* are low.

A better understanding of the mechanisms involved in reducing gene expression levels of the *BRCA1* gene or in its silencing is of fundamental importance, since the expression levels may influence the response to various agents, and also serve as therapeutic targets such as the new generation of target specific agents, namely PARP (poly ADP ribose polymerase) inhibitors.

In conclusion, it is worth noting that carriers of germline mutations in the *BRCA1* gene have vital cumulative risk much higher than the population for the development of cancer. Because of that, the knowledge of which gene is altered and the mechanisms involved enables a significant improvement in the identification of individuals at-risk, as well as in decisions about the management of risk and preventive strategies, risk reduction (prophylactic surgery and chemoprevention) and therapeutics to be offered. Through the literature reports, along with our data, we can infer that a significant portion of patients at risk for hereditary breast cancer (mutated or not in *BRCA1*) show a reduction in gene expression levels of *BRCA1*, requiring more studies to elucidate these findings. Still, we can infer that germline mutation and presence of *BRCA1* hypermethylation are mutually exclusive events and both contribute to the *BRCA*-ness. Furthermore, the presence of a constitutive epimutation should be taken into consideration in a family at high risk for hereditary breast cancer without any germline mutation identified, since the presence of this epigenetic alteration can constitute the explanation for the family history identified.

## MATERIALS AND METHODS

### Subjects

The study analyzed a convenience group of 85 women at-risk for hereditary breast cancer from the Oncogenetics Department of Barretos Cancer Hospital (BCH). In addition, 3 patients with TN tumors and presence of a germline *BRCA1* mutation from the International Research Center AC Camargo were included. Those women were referred for *BRCA1/2* genetic testing due to the presence of clinical criteria for HBOC (Hereditary Breast and Ovarian Cancer Predisposition Syndrome). For the purpose of the present study, these patients were grouped according to the genetic test results: 1) *BRCA1*-pathogenic group: 23 women (families) with personal and family history of breast cancer carrying a deleterious mutation in the *BRCA1* gene; 2) *BRCA1*-VUS group: 22 women (families) with personal and family history of breast cancer with unknown clinical significance variants (VUS) identified in the *BRCA1* gene and, 3) *BRCA1*-WT group: 43 women (families) with personal and family history of breast cancer without deleterious mutation and VUS identified in the *BRCA1* gene.

For VUS classification (and inclusion in the *BRCA1*-VUS group) the following databases were considered: Clinvar, BIC (Breast Cancer Information Core), HGMD (Human Genome Mutation Database) and LOVD IARC (Leiden Open Variation Database).

This study was approved by the Ethics Committees of the two participating Institutions, Barretos Cancer Hospital and AC Camargo Cancer Center (Process numbers: 801/2014 and 1746/13, respectively).

### Laboratory analyses

### Analyses of mutations in *BRCA1* and *BRCA2*

The analyses of the presence of germline mutations in *BRCA1* and *BRCA2* was conducted at the Center of Molecular Diagnosis of BCH as part of routine care through NGS sequencing (using the *BRCA1/2* Ampliseq panel in the Ion Torrent PGM platform) as described elsewhere by Palmero and collaborators [35]. Basically, libraries containing the PCR product of 14 multiplex PCRs were pooled, purified and fragmented according to the supplier protocol. In the sequence the libraries with the adaptors were amplified by emulsion PCR, enriched and sequenced using the Ion 316 chips, which allow the simultaneous analysis of 12 patients per chip. Data analysis was performed using *DNAstar Lasergene 10 software*, with the following parameters: (1) Q call > = 40; (2) depth of coverage > = 100 and, (3) SNP % > = 23. All the identified variants were confirmed in a new PCR reaction followed by conventional bi-directional sequencing (Sanger).

For the screening of large rearrangements over the *BRCA1* and *BRCA2* genes, Multiplex Ligation-dependent Probe Amplification Kits (MLPA) were used.

### Biological samples

For methylation and gene expression analysis a representative section of each FFPE normal and tumor tissue were stained by hematoxylin and eosin and evaluated by a pathologist to verify normal and tumor content (> 60% tumor) and macrodissected. For DNA extraction the DNeasy Blood and Tissue kit (Qiagen) was used following the manufacturer instructions. For RNA extraction, Recover All Total Nucleic Isolation Optimized for FFPE Samples (Ambion by Thermo Fisher Scientific) kit was used.

### Analyses of the quality and integrity of the extracted DNA

To verify the quality and integrity of the extracted DNA a multiplex PCR reaction with four pairs of primers for the *GAPDH* gene was performed as described by Van Beers *et al*. [36].

Basically, the PCR was performed with a final volume of 30 μL, containing 1.5 mM MgCl2; 0.2 mM dNTP (*Invitrogen*™); 0,133 μM of each primer; 1 U Taq DNA polymerase (*Invitrogen*™) and 60 ng normal/tumor DNA. The reactions were performed in a *Veriti*^®^ thermocycler (*Applied Biosystems*) using the following amplification parameters: 94°C for 1 minute, 35 cycles of 94°C for 1 minute, 56°C for 1 minute, and 72°C for 3 minutes. Finally, a final extension at 72°C for 7 minutes, finishing at 15°C. The successful amplification of DNA was checked by agarose gel electrophoresis on 1.5% *GelRed* ™ stained, visualized under UV light and documented.

### Treatment with sodium bisulfite

For methylation analysis, the DNA obtained was subjected to treatment with sodium bisulfite using *EpiTect bisulfite Kit* (*Qiagen*) and following the manufacturer's specifications. Briefly, 1 μg of DNA was mixed with 85 μl of bisulfite mix (provided in the kit), 35 μl protect DNA buffer and water to a final volume of 140 μl and placed in a thermocycler for bisulfite conversion. Next, the converted DNA was purified by several washing steps, eluted in 20 μl of ultra-pure water (*Milli-Q*) and stored at −80°C until use.

### qMSP analysis

Bisulfite-modified DNA was used as a template in fluorogenic qMSP assays carried out in a final volume of 20 μL in a 7500 Real Time PCR System (*Life Technologies*). PCR was done in separate wells for each primer/probe set and each sample was run in triplicate. The final reaction mixture contained 3 μL of bisulfite-modified DNA, 1.2 μM of forward and reverse primers, 200 nM of probe, 0.6 U of platinum Taq polymerase (*Life Technologies*), 200 μM of dNTPs, 16.6 mM of ammonium sulfate, 67 mM of Tris-HCl pH 8.0, 6.7 mM of magnesium chloride, 10 mM of mercaptoethanol, 0.1% DMSO and 1× ROX dye (*Life Technologies*). PCR was conducted with the following conditions: 95°C for 2 minutes, followed by 45 cycles at 95°C for 15 seconds and 60°C for 1 minute.

Each plate included DNA samples, multiple water blanks and serial dilutions (90–0.009 ng) of a positive control for constructing the calibration curves. Leukocyte DNA from a healthy individual was methylated *in vitro* with SssI methyltransferase (*New England Biolabs Inc*) to generate completely methylated DNA at all CpG and used as a positive control. Additionally, the level of methylation of normal breast tissue was measured in two cell lines derived from normal tissue (HB4a and MCF10A).

Primers and probes that specifically amplify the promoter regions of the *BRCA1* gene and the internal reference gene, *ACTB* were selected from the literature [37]. Samples were considered methylated when detecting amplification of at least two of the triplicates. The absence of amplification or amplification of only one of the triplicates indicates that the sample is non-methylated. The percentage of methylation on each sample was obtained by the equation: mean number of methylated copies of target gene/average number of copies of *ACTB* X 100. Cases were scored as positive if a percentage value of ≥ 4.0% was obtained, according to studies published in the literature [38, 39].

### Analyses of the assessment and quality of total RNA

Quantification of total RNA samples was first performed with the spectrophotometer *NanoDrop 2000 Spectrophotometer* (*Thermo Scientific*) by analyzing the absorbance at 260 nm. Then, to ensure a more accurate quantification, a fluorescent method with *Qubit* equipment (*Invitrogen*) was used. The analyses of the quality/integrity of total RNA was performed by electrophoresis equipment microfluidics *Bioanalyzer 2100* (*Agilent Technologies*) using *RNA Pico Chip* (*Agilent Technologies*) according to the manufacturer's recommendations.

### cDNA synthesis and real-time PCR

To obtain the cDNA, RT-PCR was performed using the *High-Capacity kit* (*Applied Biosystems*) according to the conditions provided by the manufacturer. Basically, 1 μg of total RNA was added to 2,0 μL RT buffer (10X), 0,8 μL dNTPs (100 mM), 2 μL RT Random Primers (10X), 1 μL-MultiScribe Reverse Transcriptase and RNase-free water to a volume end of 10 μL. The reactions were maintained at 25°C for 10 minutes, 37°C for 120 minutes and ended by an incubation of 5 minutes at 85°C. The reactions were performed in a *Proflex Thermal Cycler* (*Thermo Fisher Scientific*). Upon completion of synthesis, all cDNAs were kept at −20°C until use.

The real time PCR reactions were carried out in the StepOne Real-Time PCR Systems (Applied Biosystems) using the *TaqMan system* (*Applied Biosystems*). Inventoried trials “PrimeTime qPCR assay tests” (*Integrated DNA Technologies*) and TaqMan (*Applied Biosystems*) were used. Probes were used for two endogenous housekeeping genes: *ACTB* (Assay ID: Hs.PT.39a.22214847) and *GAPDH* (Assay ID: Hs.PT.39a.22214836) and for the testing gene *BRCA1* (Assay ID: Hs01556193_m1). For reactions we used 2,5 μL water, 5 μL of TaqMan Fast Advanced 2X Master Mix (*Applied Biosystems*), 0,5 μL TaqMan Gene Expression Assay 20X (*Applied Biosystems*) containing primers and probe specific for each of the genes and 2 μL of each cDNA sample in a final volume of 10 uL reaction. The PCR was conducted in 40 cycles, according to the following protocol: 2 minutes at 50°C, 20 seconds at 95°C, 1 second at 95°C, 20 seconds at 60°C. Small experimental variations in pipetting and reaction efficiency were diluted by conducting experiments in triplicate. To achieve quantification, we used the average of the CT values of triplicates. The standard expression of the gene of interest wascalculated by the mathematical model 2^-ΔCT^ [40]. The value of the median 2^- ΔCT^ obtained for all samples was used as the cutoff point for classification of samples in low and high expression. The expression levels of the *BRCA1* gene (ΔCT) were represented by the mean value of triplicates normalized by the average expression of endogenous (*GAPDH* and *ACTB*). Lower ΔCT values indicates greater expression. The value of the median ΔCT (0.55636) was used as the cutoff between high and low expression, e.g, the samples were compared independently.

### Statistical analyses

Statistical analysis was performed using SPSS v.19.0 software for Windows (Chicago, IL). Categorical variables were described by absolute frequencies and percentages relative frequencies. Quantitative variables were described by mean and standard deviation when they had symmetrical distribution and the median, minimum and maximum when they ha asymmetric distribution. The significance level for all tests was 5%.

To compare the clinical and molecular characteristics we used Chi-square test (or Fisher's exact test). Comparisons between methylated vs. mutated groups, as well as among presence vs. absence of expression of *BRCA1* in relation to clinical and molecular characteristics were also performed using the Chi-square test.
